# An international simulated-use study to assess nurses’ preferences between two lanreotide syringes for patients with neuroendocrine tumours or acromegaly (PRESTO 3)

**DOI:** 10.1007/s40618-023-02158-5

**Published:** 2023-08-08

**Authors:** D. Ferone, W. Martin, J. Williams, A. Houchard, C. Pommie, A. Ribeiro-Oliveira, A. B. Grossman

**Affiliations:** 1 Endocrinology, IRCCS Ospedale Policlinico San Martino, Genoa, Italy; 2 Department of Internal Medicine and Medical Specialties, University of Genova, Genoa, Italy; 3 Kings College Hospital Foundation Trust, ENETS Centre of Excellence, London, UK; 4 Oregon Health and Science University Pituitary Center, Portland, OR USA; 5 Ipsen, Boulogne-Billancourt, France; 6 Ipsen, Cambridge, MA USA; 7 Green Templeton College, University of Oxford, Oxford, UK; 8 Neuroendocrine Tumour Unit, ENETS Centre of Excellence, Royal Free Hospital, London, UK; 9 Centre for Endocrinology, Barts and the London School of Medicine, London, UK

**Keywords:** Syringe attributes, Nurse preference, Somatostatin analogue, Acromegaly, Neuroendocrine tumours

## Abstract

**Purpose:**

PRESTO 3 evaluated nurses’ preference for the Somatuline® Autogel® syringe versus the Lanreotide Pharmathen syringe after injection-pad testing.

**Methods:**

This international simulated-use study included oncology/endocrinology nurses with ≥ 1 years’ experience in managing neuroendocrine tumours (NETs) and/or acromegaly. Each nurse tested both syringes twice in a randomised order before completing an electronic survey. The primary objective was to assess overall preference (%, 95% confidence interval [CI]) for the Somatuline Autogel syringe versus the Lanreotide Pharmathen syringe. Secondary objectives included rating syringe performance and ranking the importance of syringe attributes.

**Results:**

Ninety-four nurses were enrolled: mean age, 41.0 (SD, 11.5) years. The percentage of nurses stating a preference (“strong” or “slight”) for the Somatuline Autogel syringe (86.2% [95% CI 77.5–92.4%]) was significantly higher than 50% (*p* < 0.0001). Performance rating was significantly higher for the Somatuline Autogel syringe versus Lanreotide Pharmathen syringe for 10 of the 11 attributes tested (*p* < 0.05). The syringe attributes considered most important when injecting patients in routine clinical practice were “easy to use from preparation to injection” (30.9%) and “comfortable to handle during use from preparation to injection” (16.0%). The attribute most commonly rated as least important was “fast administration from preparation to injection” (26.6%).

**Conclusion:**

Nurses strongly preferred the user experience of the Somatuline Autogel syringe over the Lanreotide Pharmathen syringe. “Ease of use” and “comfortable to handle” were the most important syringe attributes, and performance rating was significantly higher with Somatuline Autogel versus Lanreotide Pharmathen syringe for all but one attribute.

**Graphical abstract:**

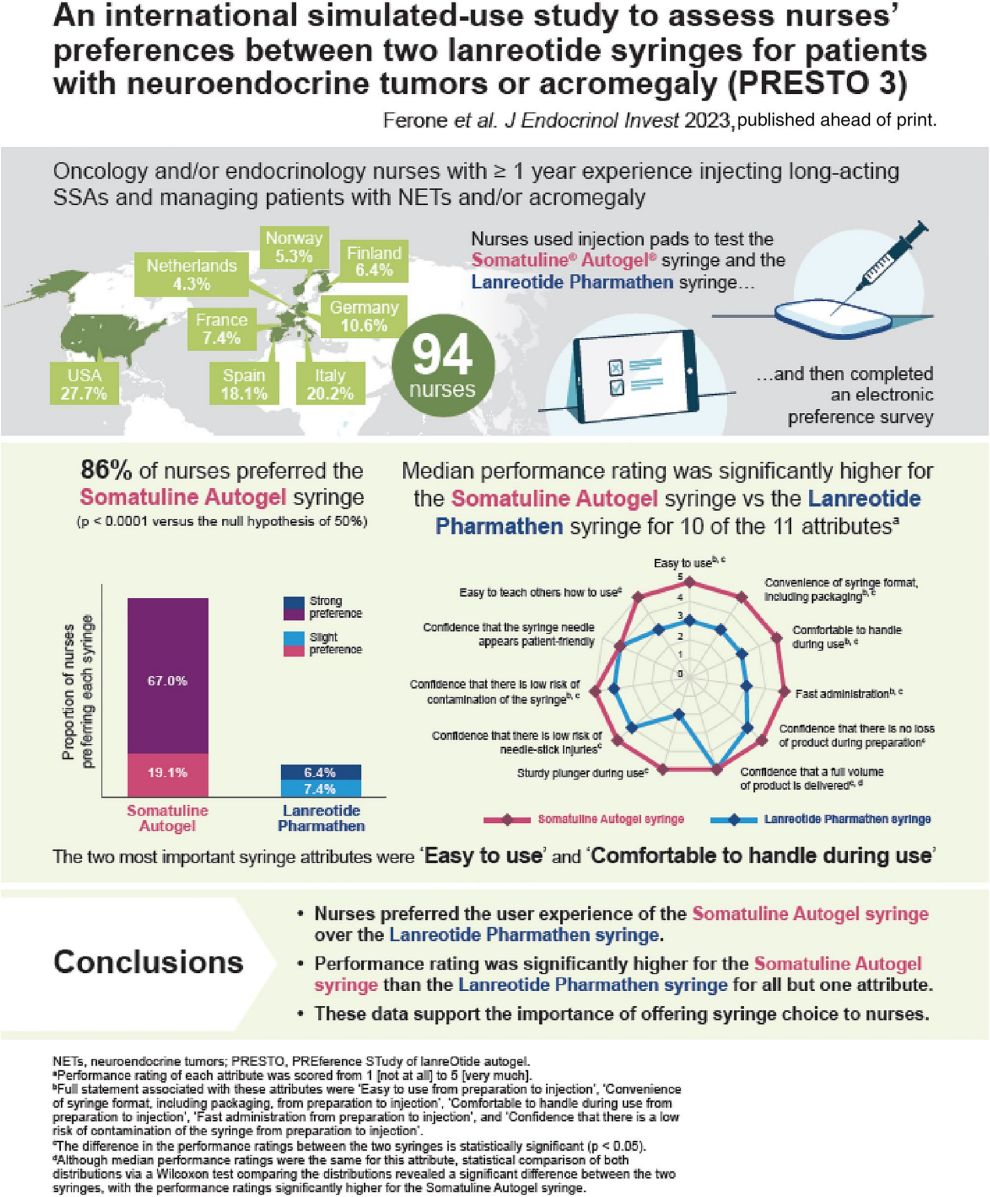

**Supplementary Information:**

The online version contains supplementary material available at 10.1007/s40618-023-02158-5.

## Plain language summary

Drugs called somatostatin analogues (SSAs) can be used to treat patients with neuroendocrine tumours or acromegaly over a prolonged period of time. SSAs are given as injections and act by slowing the production of hormones by the body and in some cases reducing the growth of the tumour. To help to provide the best care possible, it is important that the syringe used for the injection is easy to use and delivers the SSA effectively. Somatuline Autogel is a syringe that can be used to inject an SSA called lanreotide. Previous studies showed that patients and nurses preferred the injection experience when using the Somatuline Autogel syringe compared with a syringe used to inject another SSA called octreotide long-acting release. A new syringe used for lanreotide injections has been developed recently by a company called Pharmathen. In the PRESTO 3 study, we compared the user experience of the Somatuline Autogel syringe and the Lanreotide Pharmathen syringe. We asked 94 nurses from Europe and the US to test both syringes, in a randomised order, using injection pads, and then to answer questions about their overall preference between the two syringes and how well the syringe performed for a set of syringe features. Overall, 86% of nurses preferred the Somatuline Autogel syringe over the Lanreotide Pharmathen syringe. Of the 11 features of the syringe that we assessed, 10 were rated higher for the Somatuline Autogel syringe than the Lanreotide Pharmathen syringe. The syringe features “ease of use” and “comfortable to handle” were considered the most important. The results of the PRESTO 3 study indicated that there is a difference in the user experience between the syringes, particularly for confidence and ease of use, and that it is important to offer syringe choices to nurses who are using SSA injections to treat patients.

**Supplementary Information:**

The online version contains supplementary material available at 10.1007/s40618-023-02158-5.

## Introduction

Patients living with neuroendocrine tumours (NETs) and acromegaly typically require long-term medical treatment. Because of this, it is important that patients have access to easy and efficient therapeutic approaches to optimise their treatment experience and minimise the negative impact of treatment on their lives, as well as to maximise treatment adherence and persistence to medication. Somatostatin analogues (SSAs) represent the mainstay of medications used to treat NETs and acromegaly [1–3]. SSAs such as octreotide long-acting release [4, 5] and lanreotide autogel [6–9] depot formulations are common treatment options for patients with NETs and acromegaly.

Through a collaboration with patients, caregivers, and healthcare professionals, a new lanreotide autogel/depot syringe was developed to ensure that the device was more robust, ergonomic, and user-friendly (version 3.0, developed by Ipsen in 2019; available as Somatuline, Somatuline Depot and Ipstyl depending on country, and referred to as Somatuline Autogel throughout this article) [10]. Somatuline Autogel is supplied as a prefilled syringe in a ready-to-use formulation. Previous studies have shown that the Somatuline Autogel syringe was preferred by the majority of nurses [11], and patients were less likely to experience prolonged pain or technical issues with the associated device [12], compared with the latest octreotide long-acting release syringe (Novartis).

The Pharmathen syringe, used to deliver generic lanreotide for patients with gastro-enteropancreatic NETs and acromegaly, is now also available for depot injection in the US (*Cipla* Lanreotide Injection) and Europe (Advanz generic lanreotide brand names: *Mytolac*, *Myrelez*, *Myrelez L.P.*, and *Mytolente*) [8, 9]. The Lanreotide Pharmathen syringe is supplied as a prefilled syringe with a separate needle and requires assembly before use [8, 9].

To date, no studies have evaluated the Somatuline Autogel syringe versus the Lanreotide Pharmathen syringe. PRESTO 3 was a simulated-use study designed to evaluate nurses’ preference for the Somatuline Autogel syringe versus the Lanreotide Pharmathen syringe among nurses experienced in treating patients with NETs and/or acromegaly.

## Methods

### Study design and procedures

PRESTO 3 was an international prospective simulated-use study evaluating the overall preference of nurses between the Somatuline Autogel and Lanreotide Pharmathen syringes. Nurses were recruited from various organisations and institutions (Centres of Excellence, hospital and community health services, and nurse networks) across Europe and the United States (US). Each region (Europe and the US) was required to contribute at least 25% of the participating nurses to avoid a substantial imbalance. No nurses who participated in the first PRESTO study [11] were included in PRESTO 3.

**Fig. 1:**
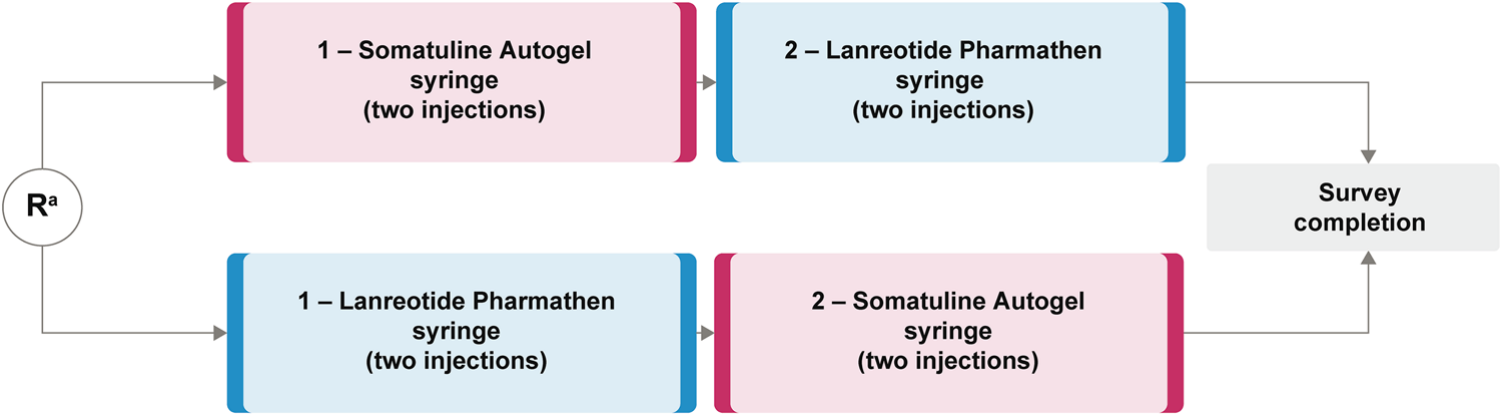
Study design. ^a^Nurses were randomly allocated to the order in which they tested the syringes. This randomisation was performed within a session with a block size of two. *R* randomisation

**Fig. 2:**
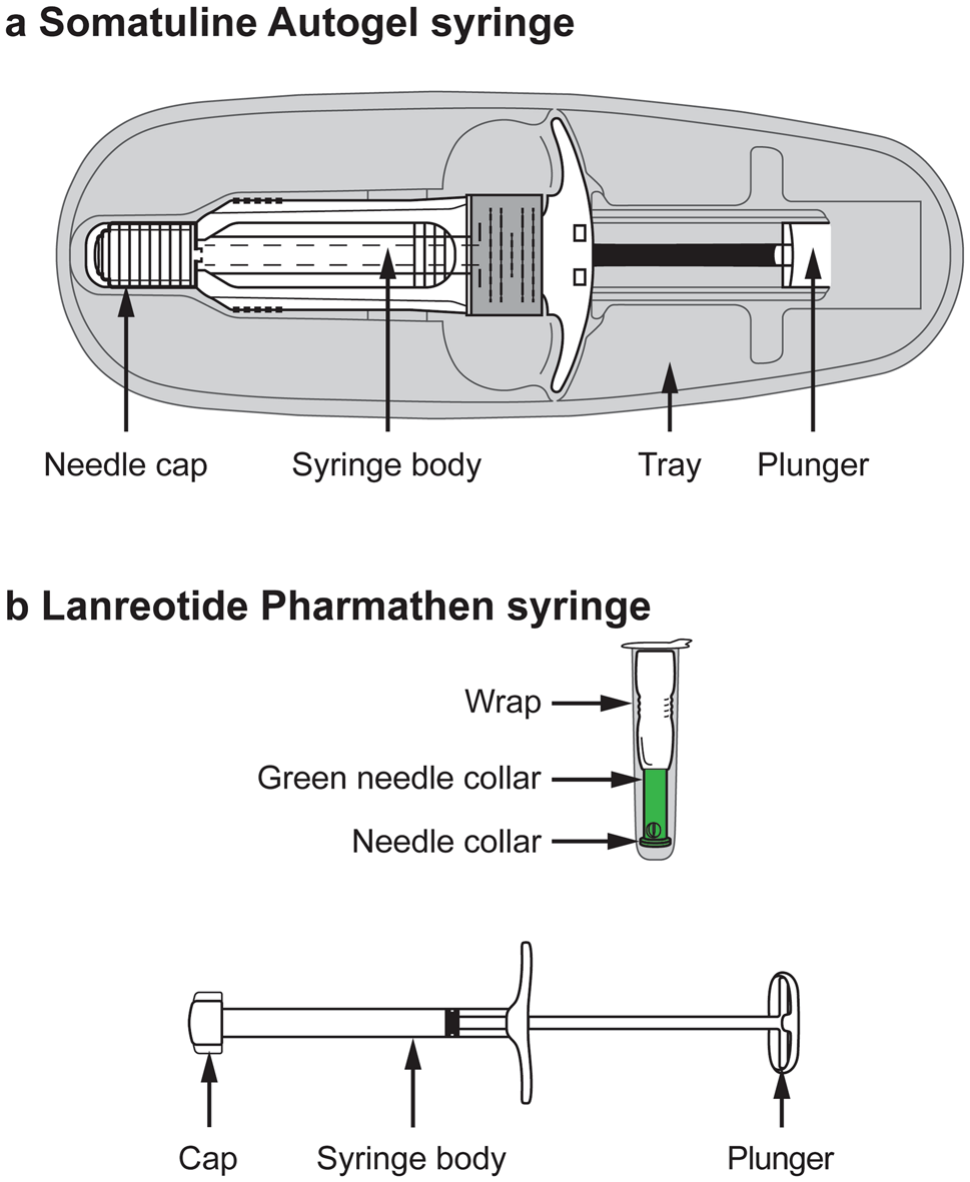
Somatuline Autogel syringe (**a**) and Lanreotide Pharmathen syringe (**b**) package contents. Somatuline Autogel syringe: prefilled, ready-to-use syringe, fitted with an automatic safety system. Each syringe is packed in a laminate pouch and a carboard box containing one 0.5 ml syringe with a 1.2 mm × 20 mm needle. Lanreotide Pharmathen syringe: Semi-transparent plastic syringe with a single-use needle device with a needle shield covering all or part of the needle (depending on the exact version of the syringe used). Each syringe is packed in an aluminium pouch and box containing one 0.5 ml syringe with a co-packaged 1.2 mm × 20 mm safety needle. Source: Mytolac Package Leaflet (March 2021) and Ipsen internal documentation ("SmPC of Mytolac® & Somatuline Autogel®”, from Global Regulatory Affairs, Global Medical Affairs and Global Marketing, dated September 9 2021)

Nurses were assigned to a syringe testing and survey session (approximately 5–10 nurses per session), which was conducted in the local language. The session lasted approximately 2 h including arrival, presentation of session, training, syringe testing, and survey completion. Each nurse tested both syringes twice in a randomised order, before completing an electronic survey (Fig. 1). Somatuline Autogel is a prefilled syringe that is ready-to-use, fitted with an automatic safety system, and is packaged in a laminated pouch and a cardboard box (Fig. 2a). The Lanreotide Pharmathen syringe is supplied as a filled syringe that is accompanied by a single-use needle device with a needle shield covering all or part of the needle (depending on the exact version of the syringe used), and is packaged in an aluminium pouch and a carton box (Fig. 2b).

Information was recorded on nurses’ overall syringe preference, the performance of each syringe with regard to 11 attributes, and their selection of the two most important and the least important attribute. The 11 attributes assessed were (Table 1):

- Easy to use from preparation to injection
- Convenience of syringe format, including packaging, from preparation to injection
- Comfortable to handle during use from preparation to injection
- Fast administration from preparation to injection
- Confidence that there is no loss of product during preparation
- Confidence that a full volume of the product is delivered
- Sturdy plunger during use
- Confidence that there is a low risk of needle-stick injuries
- Confidence that there is a low risk of contamination of the syringe from preparation to injection
- Confidence that the syringe needle appears patient-friendly
- Easy to teach others how to use

**Table 1:** Syringe attributes assessed for both syringes

Attributes assessed
1. Easy to use from preparation to injection
2. Convenience of syringe format, including packaging, from preparation to injection
3. Comfortable to handle during use from preparation to injection
4. Fast administration from preparation to injection
5. Confidence that there is no loss of product during preparation
6. Confidence that a full volume of the product is delivered
7. Sturdy plunger during use
8. Confidence that there is a low risk of needle-stick injuries
9. Confidence that there is a low risk of contamination of the syringe from preparation to injection
10. Confidence that the syringe needle appears patient-friendly
11. Easy to teach others how to use

The syringe attributes to be assessed in this study were defined internally by the study team in line with the PRESTO study [11], but were then modified slightly in response to discussions with two representative nurses who are authors on this publication to reflect the fact that, unlike in PRESTO, the current study compared two pre-filled syringes (J Williams from the US who has experience in acromegaly, and W Martin from the UK who has experience in NETs). Any difficulties with the Somatuline Autogel syringe and/or Lanreotide Pharmathen syringe that were experienced during the simulated injection were also recorded. Data were pseudo-anonymised to protect nurses’ confidentiality.

### Nurses enrolled in the study

Nurses that were enrolled in the study were oncology or endocrinology nurses experienced in treating patients with NETs and/or acromegaly for at least 1 year. Eligible nurses were responsible for administering treatment and had at least 1 year of experience in injecting long-acting SSAs (with at least two different types of syringe), had treated at least four patients with NETs or acromegaly per year or at least one patient every 3 months, and were practising in settings treating patients with long-acting SSAs. All nurses signed a consent form for both syringe testing and data collection prior to participation. During the study, the percentage of nurses with experience injecting with the Lanreotide Pharmathen syringe was monitored to ensure that at least 25% of nurses had experience with this syringe.

### Objectives

The primary objective was to assess the nurses’ overall preference for the Somatuline Autogel and the Lanreotide Pharmathen syringes after injecting into injection pads. The primary endpoint was the percentage of nurses with an overall preference for either the Somatuline Autogel syringe or the Lanreotide Pharmathen syringe after injection pad testing.

Secondary objectives included: (a) describing the socio-demographics and clinical characteristics (e.g., experience) of nurses, (b) describing the attribute performance for each syringe, (c) describing the importance of each attribute, and (d) assessing how attributes and factors relate to nurses’ preferences.

Analyses of the primary and secondary objectives were repeated for subgroups based on region (US versus Europe).

### Statistics

The sample size calculation was based on an exact test for a binomial proportion with a two-sided 5% alpha level and a power of 80%. Assuming an expected proportion at 65%, the null hypothesis being that the proportion equals 50%, 92 nurses were planned to be recruited. Analyses were conducted on the main study population which included all nurses enrolled in the study who: (a) provided informed consent in the survey, (b) were assigned a randomisation number, (c) completed the testing session with the two syringes, (d) assessed their preference for the Somatuline Autogel syringe compared with the Lanreotide Pharmathen syringe, (e) met all eligibility criteria, and (f) had no major data quality concerns.

For the primary endpoint, the percentage (95% exact [Clopper–Pearson] confidence interval [CI]) of nurses preferring the Somatuline Autogel syringe (“strong” or “slight” preference) or the Lanreotide Pharmathen syringe was calculated. A one-sample exact binomial test of proportions was used to test whether the preference for the Somatuline Autogel syringe was different to 50% (the null hypothesis was that the preference was equal to 50%).

For secondary endpoints, descriptive summaries of the socio-demographics (age and gender) and clinical experiences (area of clinical practice, type of patients injected, setting, length of experience, and number of patients injected using each syringe) were provided. The performance rating for each attribute (1 = Not at all, 2 = A little bit, 3 = Somewhat, 4 = Quite a bit, and 5 = Very much) was described for each syringe and compared using a Wilcoxon signed-rank test. The two most important and the least important attributes were also described.

Variables associated with nurses’ overall preference for the Somatuline Autogel syringe, including the performance of each attribute (based on the difference between syringes in the performance rating for each attribute) and demographic factors (region, age, gender, area of clinical practice, setting used to inject patients, and experience injecting long-acting SSAs), were identified via univariable logistic regression models. Potential predictors that were significantly associated with the preference for Somatuline Autogel at a significance level of 0.2 were included in the final stepwise selection multivariable logistic regression model after checks of multicollinearity.

### Ethics

The survey was conducted in compliance with relevant regulations, including the Epidemiological Studies published by the Council for International Organizations of Medical Sciences [12]. Institutional Review Board review was not required due to the design of the study (simulated-use study and no involvement of patients).

## Results

### Enrolled nurse characteristics

In total, 94 nurses were enrolled in the study, all of whom completed the testing session and provided their preference for the Somatuline Autogel syringe compared with the Lanreotide Pharmathen syringe (Table 2). The overall mean (standard deviation [SD]) age was 41.0 (11.5) years and 84% of nurses were female. Across regions, 72.3% were enrolled from Europe and 27.7% from the US. Oncology was the most common area of clinical practice (72.3%), followed by endocrinology (18.1%). The nurses were experienced in injecting patients with NETs (64.9%), acromegaly (7.4%), or both (27.7%), and in using the Somatuline Autogel syringe (90.4%), the Lanreotide Pharmathen syringe (48.9%), and octreotide long-acting release (73.4%) or generic (43.6%) octreotide syringes. Twenty-one sessions were conducted with a mean (SD) of 4.5 (2.8) nurses per session.

**Table 2:** Nurse demographic characteristics and clinical experience

	Nurses included in analysis (*N* = 94)
**Age, years, mean (SD)**	41.0 (11.5)
**Gender,** **n** **(%)**	
Female	79 (84.0)
Male	14 (14.9)
Prefer not to say	1 (1.1)
**Region/country, n** **(%)**	
Europe	68 (72.3)
Finland	6 (6.4)
France	7 (7.4)
Germany	10 (10.6)
Italy	19 (20.2)
Netherlands	4 (4.3)
Norway	5 (5.3)
Spain	17 (18.1)
United States	26 (27.7)
**Type of patients injected,** **n** **(%)**	
NETs	61 (64.9)
Acromegaly	7 (7.4)
Both	26 (27.7)
**Device experience**, **n** **(%)**^a^	
Somatuline Autogel	85 (90.4)
Lanreotide Pharmathen syringe	46 (48.9)
Octreotide LAR	69 (73.4)
Generic octreotide	41 (43.6)
**Main area of clinical practice,** **n** **(%)**	
Oncology	68 (72.3)
Endocrinology	17 (18.1)
Gastroenterology	5 (5.3)
Other	4 (4.3)
**Setting used to inject patients, n** **(%)**	
Centre of Excellence	46 (48.9)
Local hospital	34 (36.2)
Other	14 (14.9)

*LAR* long-acting release, *NET* neuroendocrine tumour, *SD* standard deviation

^a^Multiple response question

### Primary endpoint: overall preference for the Somatuline Autogel or Lanreotide Pharmathen syringe

**Fig. 3:**
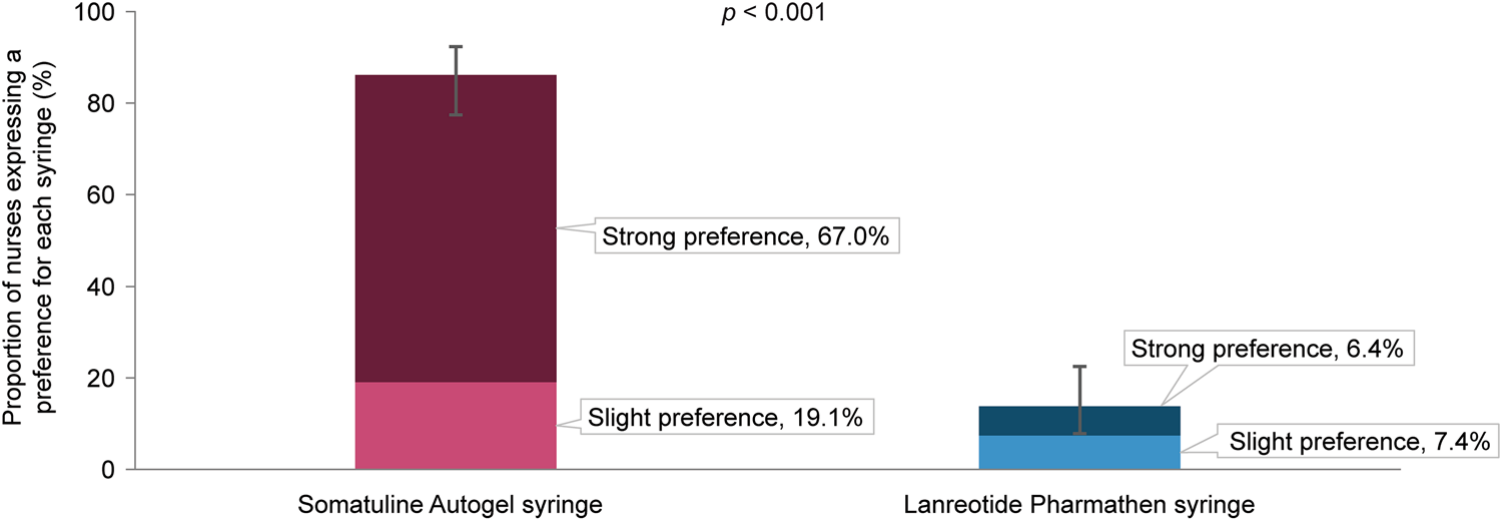
Preference for the Somatuline Autogel syringe or the Lanreotide Pharmathen syringe. Error bars indicate 95% confidence intervals

The percentage of nurses having preference (“strong” or “slight”) for the Somatuline Autogel syringe was statistically significantly higher than 50% (*p* < 0.0001). Overall, 86.2% (81/94) of nurses expressed a higher preference for the Somatuline Autogel syringe (67.0% [63/94] “strong”, 19.1% [18/94] “slight”). In contrast, 13.8% (13/94) preferred the Lanreotide Pharmathen syringe (6.4% [6/94] “strong”, 7.4% [7/94] “slight”) (Fig. 3).

### Secondary endpoints

#### Performance rating and importance ranking of attributes

**Fig. 4:**
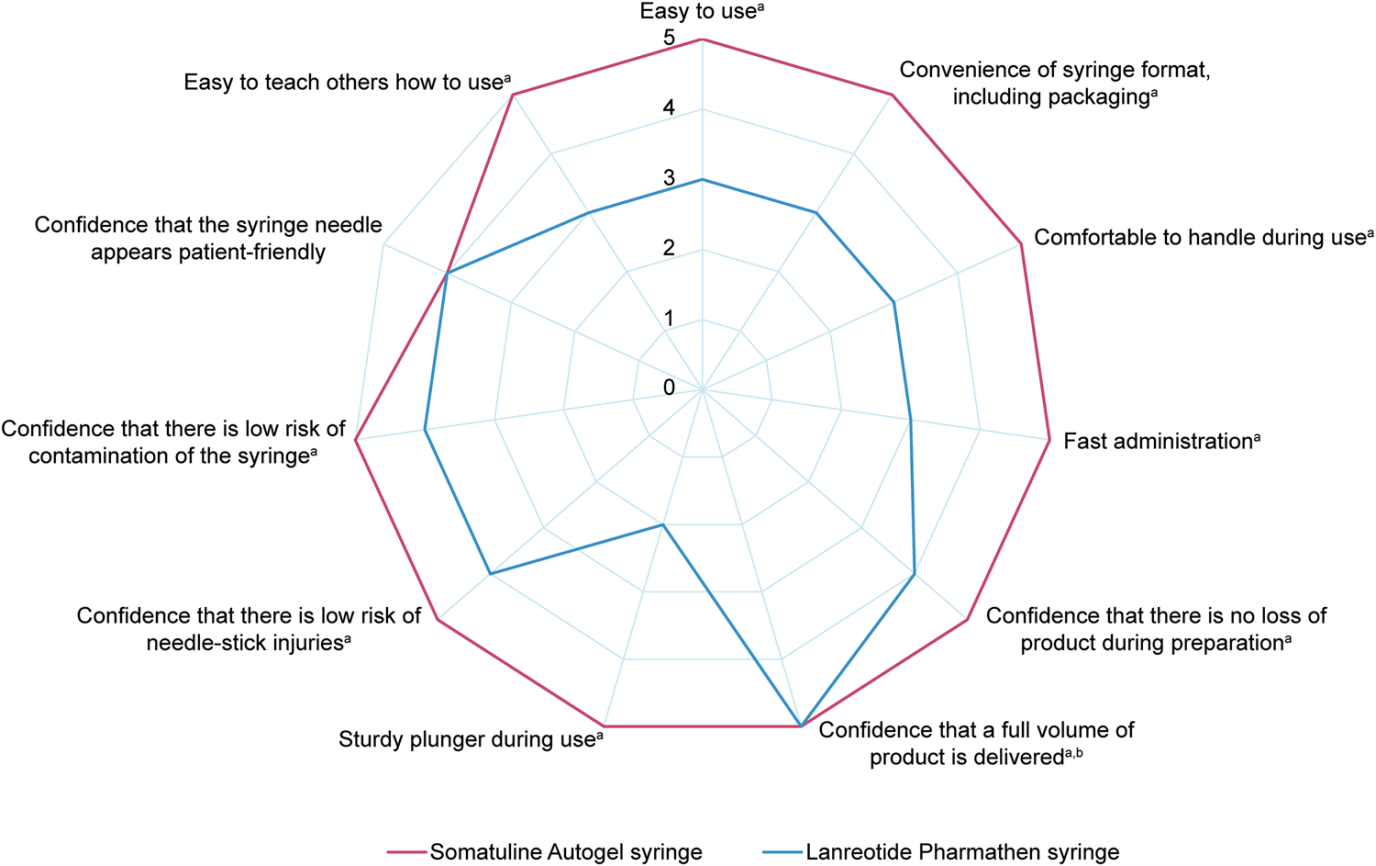
Median performance ratings of attributes for Somatuline Autogel Syringe and Lanreotide Pharmathen Syringe. Comparison between Somatuline Autogel syringe and Lanreotide Pharmathen syringe was conducted using the paired Wilcoxon signed-rank test. ^a^The difference in the performance ratings between the two syringes is statistically significant (*p* < 0.05). ^b^Although median performance ratings were the same for this attribute, statistical comparison of both distributions via a Wilcoxon test comparing the distributions revealed a significant difference between the two syringes, with the performance ratings significantly higher for the Somatuline Autogel syringe

Performance rating was significantly higher with the Somatuline Autogel syringe than with the Lanreotide Pharmathen syringe for 10 of the 11 attributes tested (*p* < 0.05) (Fig. 4). The greatest difference in ratings was for “sturdy plunger during use” in favour of Somatuline Autogel.

**Fig. 5:**
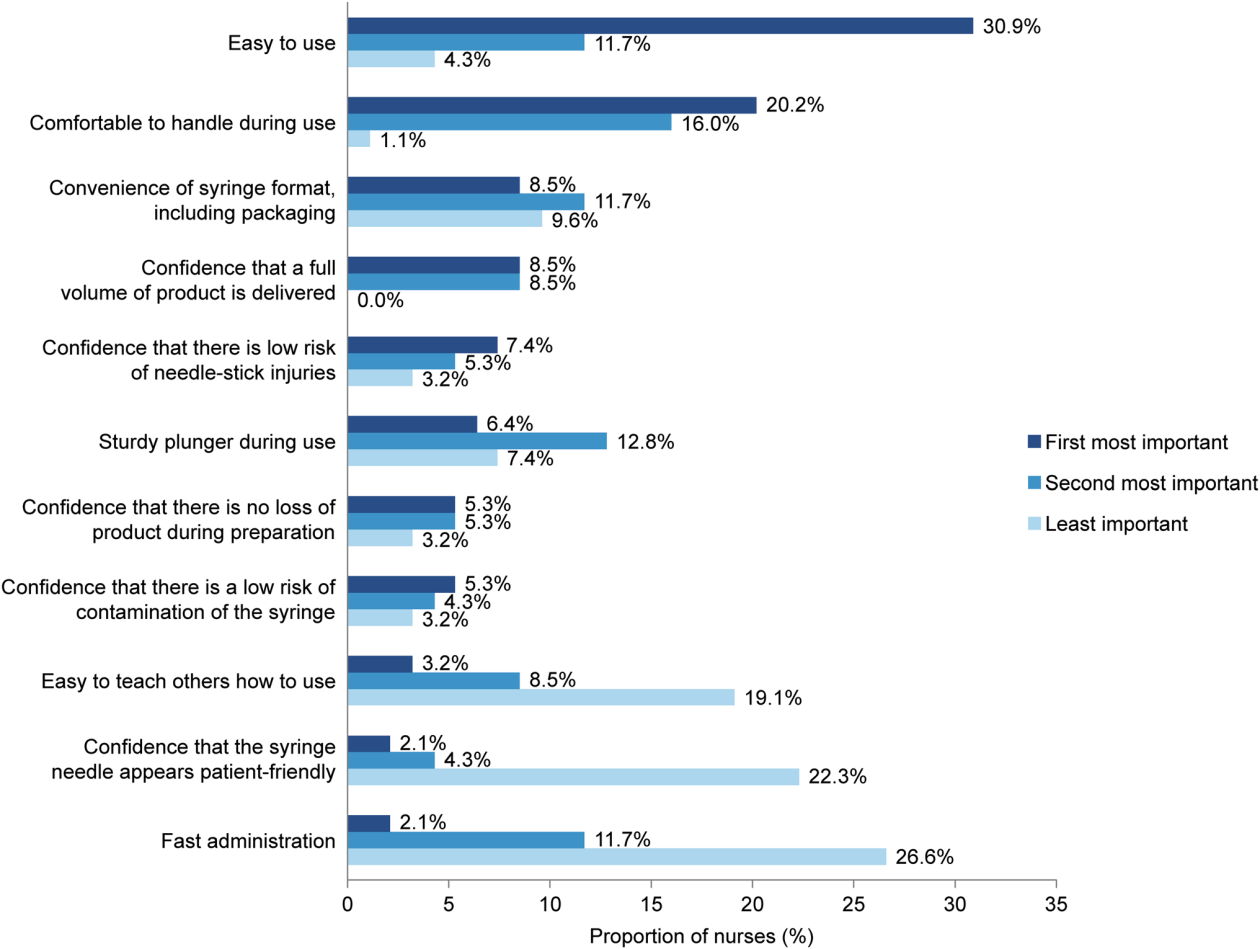
Ranking of attributes by importance

The ranking of attributes as first most important, second most important, and least important, are reported in Fig. 5. The attribute most commonly rated as the first most important was “easy to use from preparation to injection”, selected by 30.9% of nurses. The attribute most commonly rated as the second most important was “comfortable to handle during use from preparation to injection”, selected by 16.0% of nurses. The attribute most commonly rated as least important was “fast administration from preparation to injection”, selected by 26.6% of nurses.

#### Relationship between attributes/factors and syringe preference

**Fig. 6:**
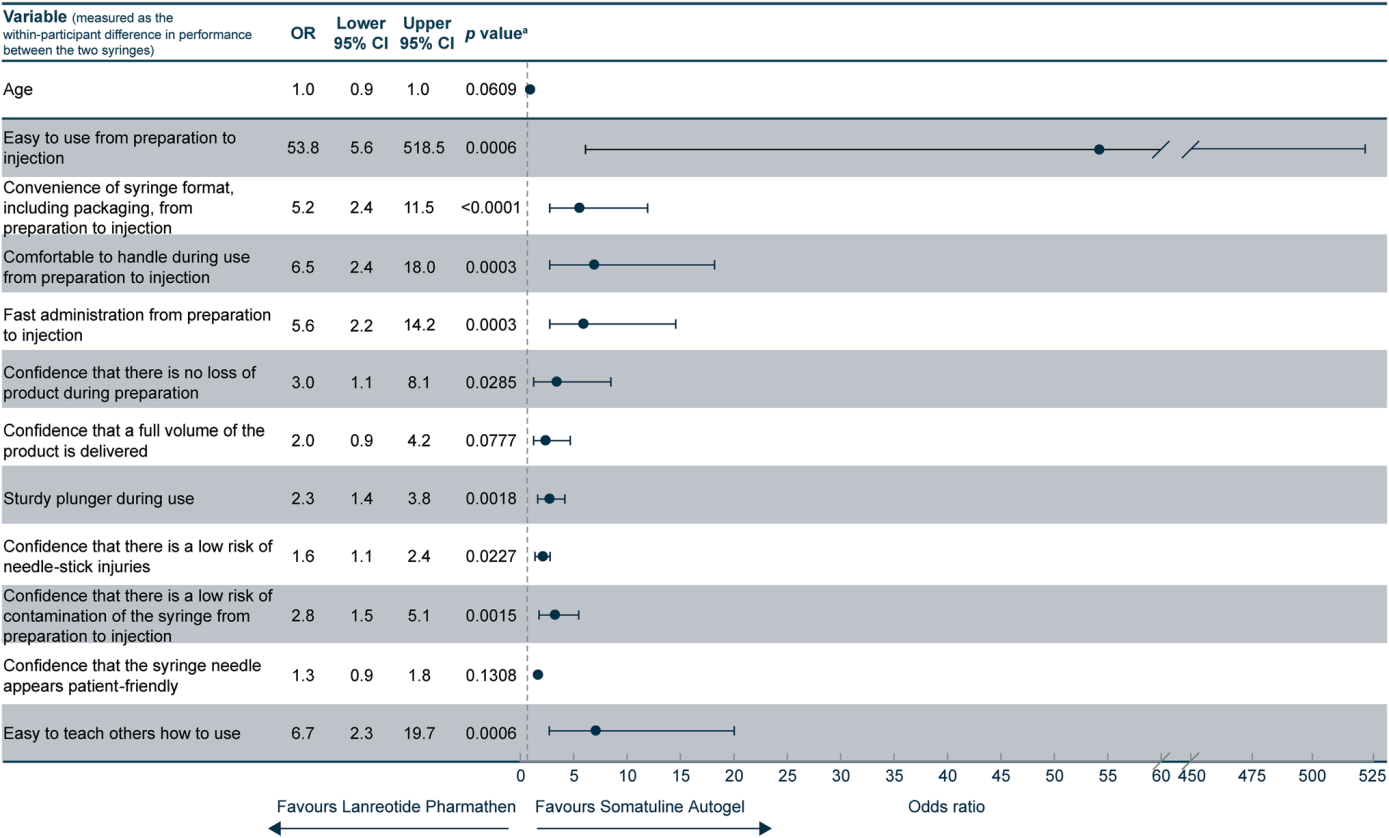
Relationship between factors/attributes and overall preference for the Somatuline Autogel syringe: univariate logistic regression model. ^a^Variables (age and syringe attributes) shown in Fig. 6 had a* p* value < 0.2 and were considered for inclusion in the stepwise selection multivariate logistic regression multivariable model. Region, gender, main area of clinical practice, settings used to inject patients and experience injecting long acting SSAs were all found to not be associated with the overall preference of nurses as per univariate logistic regression, as they did not meet the *p* < 0.2 threshold). *CI* confidence interval, *OR* odds ratio

Nurses’ age and the difference in ratings between the syringes for the 11 attributes were found to be associated with the overall preference of nurses as per univariate logistic regression (*p* < 0.2) (Fig. 6). There was no evidence of important multicollinearity because correlations were lower than 0.8. Moderate correlations were observed between some of the attributes (correlation coefficient 0.4–0.6). Based on the multivariate logistic regression model with stepwise selection, “easy to use from preparation to injection” was the only variable that remained associated with the outcome of overall nurses’ preference (odds ratio 53.8; 95% CI 5.6, 518.5; *p* = 0.0006). For every one unit increase in the difference in the performance of this attribute between the two syringes, the preference for the Somatuline Autogel syringe was 53.8 times more likely.

#### Subgroup analysis by region

In Europe and the US, nurses were experienced in injecting patients with NETs (63.2% and 69.2%, respectively), acromegaly (5.9% and 11.5%), or both diseases (30.9% and 19.2%) (Supplementary Table 1). Findings by region were consistent with the findings for the overall study population. For overall syringe preference, 85.3% of the nurses in Europe and 88.5% of the nurses in the US preferred the Somatuline Autogel syringe (*p* < 0.0001 in both regions). Regardless of the region (Europe/US), the performance of the Somatuline Autogel syringe was rated as better than the Lanreotide Pharmathen syringe for the majority of attributes (Supplementary Table 2).

The two attributes nurses considered to be the most important when injecting patients in routine clinical practice in both regions were “easy to use from preparation to injection” (Europe, 33.8%; US, 23.1%) and “comfortable to handle during use from preparation to injection” (Europe, 16.2%; US, 30.8%) (Supplementary Fig. 1a and 1b). Of all the attributes assessed, in Europe, nurses considered the least important attribute to be “fast administration from preparation to injection” (26.5%); in the US, the attributes “fast administration from preparation to injection” and “easy to teach others how to use” were both considered least important (both 26.9%) (Supplementary Fig. 1a and 1b).

#### Difficulties reported with syringes

Overall, 34% of nurses reported difficulties while using the Lanreotide Pharmathen syringe during the injection test. Difficulties were related to preparation and administration with the syringe. On the other hand, 3% of nurses reported difficulties related to preparation and administration while using the Somatuline Autogel syringe.

## Discussion

Patients with NETs and acromegaly require long-term medical treatment. Previous collaborative studies involving patients, caregivers, and other healthcare providers have highlighted that the syringe device used for the treatment of NETs and acromegaly is crucial [10–12]. Drug delivery systems have evolved to provide the optimal treatment experience to patients living with NETs and acromegaly, while simultaneously maximising treatment adherence. Meeting the needs of nurses, to ensure that they can deliver treatment easily and effectively, are expected to translate to benefits in patient care.

Advances in the design of the Somatuline Autogel syringe have been guided by input from patients, caregivers, and healthcare professionals [10], and the results of a previous simulated-use study (PRESTO) showed that the redesigned lanreotide Somatuline Autogel syringe was strongly preferred over the available octreotide long-acting release syringe by the majority of nurses [11]. The current study evaluated the preference of nurses for the Somatuline Autogel syringe versus the Lanreotide Pharmathen syringe, which has recently become available in Europe and the US [8, 9].

In this international simulated-use study, the majority (86.2%) of nurses preferred the Somatuline Autogel syringe over the Lanreotide Pharmathen syringe, with over two-thirds (67.0%) of nurses indicating a strong preference for Somatuline Autogel. A sensitivity analysis, which excluded four nurses who only had experience with one type of syringe (octreotide generic or long-acting release) or less than 1 year of experience, produced consistent results: 85.6% of nurses preferred the Somatuline Autogel syringe (*p* < 0.0001).

Ease of use and ergonomic design were clearly important to participating nurses, with the two syringe attributes considered the most important when injecting patients in routine clinical practice identified as “easy to use from preparation to injection” and “comfortable to handle during use from preparation to injection”. Overall, speed of administration was identified as least important to nurses. This may be because the same drug (lanreotide) was being injected with both syringes and the difference in injection speed between the two syringes was not enough for nurses to consider it to be clinically meaningful, rather than it not being important.

Participating nurses rated the performance of the Somatuline Autogel as significantly higher than the Lanreotide Pharmathen syringe for 10 of the 11 attributes assessed (*p* < 0.05). The only attribute that did not show a significant difference between syringes was “confidence that the syringe needle appears patient-friendly”. Based on the multivariate logistic model, “easy to use from preparation to injection” was the only variable significantly associated with nurses’ syringe preference. Results showed that for every one unit increase in the difference in the performance of this attribute between the two syringes, a preference for the Somatuline Autogel syringe was more than 50 times more likely.

Previous changes to the Somatuline Autogel syringe design included: larger flanges; a larger and more rigid non-transparent needle cap; new plunger supports; and a new protective tray [10]. In the PRESTO study, comparison of this redesigned Somatuline Autogel syringe with the octreotide long-acting release syringe [11] revealed that the attributes of most importance to nurses were those related to confidence that the syringe would not become clogged and that there would be no loss of product. These specific concerns were potentially driven by the comparator, for which risk of clogging had been reported previously for the current (at the time of the PRESTO study) and previous versions of the octreotide long-acting release syringe [13–16].

In the current study, “easy to use from preparation to injection” was identified as the main driver of the nurses’ preference for the Somatuline Autogel syringe. This attribute was likely influenced by the difference between the packaging and construction of the two syringes. The Somatuline Autogel syringe is supplied ready-to-use as a prefilled syringe [10] requiring no assembly by the nurse prior to use. In contrast, the Lanreotide Pharmathen syringe has a syringe body and a separately packaged needle cap with a shield. The Lanreotide Pharmathen syringe also has a thinner plunger than the Somatuline Autogel syringe and, notably, in this study the attribute “sturdy plunger during use” displayed the greatest difference in performance ratings between syringes (favouring Somatuline Autogel). The lack of significant difference between syringes in the rating of the attribute “confidence that the syringe needle appears patient friendly” was anticipated because both syringes have a needle cap or shield that protects and covers at least part of the needle (the Lanreotide Pharmathen syringe shield is retractile). The addition of a large, ridged, non-transparent cap that fitted over the needle, instead of a see-through needle shield, was a key feature in the development of the new Somatuline Autogel syringe in 2019 [11].

Several other attributes of the Somatuline Autogel syringe that were rated more highly than the Lanreotide Pharmathen syringe have potential implications for patient care that warrant further investigation. For example, the higher rating for “easy to teach others how to use” may have implications for the suitability of the Somatuline Autogel syringe for independent injection, which may lead to cost savings as well as greater convenience and a sense of independence for patients [17, 18]. Although speculative at this stage, it is feasible that the higher rating for “sturdy plunger during use” for the Somatuline Autogel syringe than the Lanreotide Pharmathen syringe may impact the level of injection site pain experienced by patients as well as the likelihood of tissue damage leading to local injection-site reactions. Pain during and after injection, and local injection-site reactions represent a substantial burden for some patients receiving SSA injections [12]. If the nurses’ greater confidence in the Somatuline Autogel syringe in terms of “confidence that there is a low risk of contamination of the syringe from preparation to injection” and “confidence that there is a low risk of needle-stick injuries” translates into actual improvements in these areas versus the Lanreotide Pharmathen syringe, this would clearly have implications for the safety of patients and health care providers. Similarly, if nurses’ higher ratings for “confidence that there is no loss of product during preparation” and “confidence that a full volume of the product is delivered” translated into tangible improvements in lanreotide delivery with the Somatuline Autogel syringe versus the Lanreotide Pharmathen syringe, this may translate to differences in patient outcomes. However, given that no patient outcomes were included in PRESTO 3, such conclusions are purely hypothetical at this stage.

Key strengths of the PRESTO 3 study include the robust study design with randomisation, both syringes being tested twice, and representation of different regions and countries. The stringent inclusion criteria for nurses, specifically that they have at least 1 years’ experience of injecting long-acting SSAs with at least two different types of syringes, was another strength of the study, as was the inclusion of nurses practicing in multiple clinical settings. Importantly, nurses involved in the earlier PRESTO study were not included in PRESTO 3. The relative limitations of the study include the use of injecting pads, raising the possibility that these results may not transfer into clinical practice when injecting patients. Because the Lanreotide Pharmathen syringe has only recently become available, nurses had less experience using this syringe than the Somatuline Autogel syringe; however, there was no association between nurses’ syringe preference and level of experience so this is not expected to have affected the findings. In addition, although there are minor visual differences between the needle safety shield of the Pharmathen Cipla syringe (US) and the Pharmathen generic syringe (Europe), the lack of regional effects on the overall preference of nurses supports the inclusion of both US and EU Pharmathen syringes within the same arm of the study. Another limitation of the study was the open-label design. This is particularly pertinent because the study was industry funded, and thus the nurses may have perceived this as a bias. Finally, patients’ perceptions may differ from those of the nurses, and the study does not provide data on whether the attributes valued by the nurses translate to improvements in patient care.

This is the first time that nurses’ preference for the Somatuline Autogel syringe versus the Lanreotide Pharmathen syringe has been assessed. This simulated-use study demonstrated that most nurses preferred the user experience of the Somatuline Autogel syringe over the Lanreotide Pharmathen syringe. The results from PRESTO 3 confirm the difference in the confidence and ease of operation of different syringes, and illustrate the importance of offering syringe choice to nurses who are treating patients with SSA injections.

### Supplementary Information

Below is the link to the electronic supplementary material.

Supplementary file1 (DOCX 662 KB)

## Data Availability

Qualified researchers may request access to study data that underlie the results reported in this publication. Additional relevant study documents, including the clinical study report, study protocol with any amendments, annotated case report form, statistical analysis plan and dataset specifications may also be made available. Data will be anonymised, and study documents will be redacted to protect the privacy of study participants. Where applicable, data from eligible studies are available 6 months after the studied medicine and indication have been approved in the US and EU, or after the primary manuscript describing the results has been accepted for publication, whichever is later. Further details on Ipsen's sharing criteria, eligible studies and process for sharing are available here (https://vivli.org/members/ourmembers/). Any requests should be submitted to www.vivli.org for assessment by an independent scientific review board.
