# Supplementary material for: An international simulated-use study to assess nurses’ preferences between two lanreotide syringes for patients with neuroendocrine tumours or acromegaly (PRESTO 3)
Source: J Endocrinol Invest. 2023 Aug 8;47(2):421–32. doi: 10.1007/s40618-023-02158-5 (PMC10859340; doi:10.1007/s40618-023-02158-5)
Supplement: Supplementary file 1 — Supplementary file1 (DOCX 662 KB) [file 40618_2023_2158_MOESM1_ESM.docx]

Supplementary tables

Supplementary Table 1. Nurse demographic characteristics and clinical experience by region

|  | **Europe**  **(N = 68)** | **United States**  **(N = 26)** |
| --- | --- | --- |
| **Age, years, mean (SD)** | 39.1 (11.1) | 46.0 (11.3) |
| **Gender, n (%)**  Female  Male  Prefer not to say | 55 (80.9)  12 (17.6)  1 (1.5) | 24 (92.3)  2 (7.7)  0 (0.0) |
| **Region/country, n (%)**  Europe  Finland  France  Germany  Italy  Netherlands  Norway  Spain  United States | 68 (100.0)  6 (8.8)  7 (10.3)  10 (14.7)  19 (27.9)  4 (5.9)  5 (7.4)  17 (25.0)  0 (0.0) | 0 (0.0)  0 (0.0)  0 (0.0)  0 (0.0)  0 (0.0)  0 (0.0)  0 (0.0)  0 (0.0)  26 (100.0) |
| **Type of patients injected, n (%)**  NETs  Acromegaly  Both | 43 (63.2)  4 (5.9)  21 (30.9) | 18 (69.2)  3 (11.5)  5 (19.2) |
| **Device experience, n (%)***  Somatuline Autogel  Lanreotide Pharmathen syringe  Octreotide LAR  Generic octreotide | 61 (89.7)  41 (60.3)  44 (64.7)  34 (50.0) | 24 (92.3)  5 (19.2)  25 (96.2)  7 (26.9) |
| **Main area of clinical practice, n (%)**  Oncology  Endocrinology  Gastroenterology  Other | 47 (69.1)  13 (19.1)  5 (7.4)  3 (4.4) | 21 (80.8)  4 (15.4)  0 (0.0)  1 (3.8) |
| **Setting, n (%)**  Center of Excellence  Local hospital  Other | 32 (47.1)  28 (41.2)  8 (11.8) | 14 (53.8)  6 (23.1)  6 (23.1) |

*Multiple response question.

LAR, long-acting release; NET, neuroendocrine tumor; SD, standard deviation

Supplementary Table 2. Performance ratings of attributes for the two syringes, in classes and by region^a^

|  | **Europe**  **(N = 68)** | **United States**  **(N = 26)** |
| --- | --- | --- |
| **Easy to use** **from preparation to injection (%)**  Better with the Somatuline Autogel syringe  Same rating for both syringes  Better with the Lanreotide Pharmathen syringe | 85.3  7.4  7.4 | 80.8  15.4  3.8 |
| **Convenience of syringe format, including packaging from preparation to injection (%)**  Better with the Somatuline Autogel syringe  Same rating for both syringes  Better with the Lanreotide Pharmathen syringe | 85.3  5.9  8.8 | 88.5  11.5  0.0 |
| **Comfortable to handle during use from preparation to injection (%)**  Better with the Somatuline Autogel syringe  Same rating for both syringes  Better with the Lanreotide Pharmathen syringe | 85.3  8.8  5.9 | 96.2  0.0  3.8 |
| **Fast administration from preparation to injection (%)**  Better with the Somatuline Autogel syringe  Same rating for both syringes  Better with the Lanreotide Pharmathen syringe | 76.5  16.2  7.4 | 73.1  19.2  7.7 |
| **Confidence that there is no loss during preparation (%)**  Better with the Somatuline Autogel syringe  Same rating for both syringes  Better with the Lanreotide Pharmathen syringe | 54.4  45.6  0.0 | 46.2  53.8  0.0 |
| **Confidence that a full volume of the product is delivered (%)**  Better with the Somatuline Autogel syringe  Same rating for both syringes  Better with the Lanreotide Pharmathen syringe | 47.1  47.1  5.9 | 34.6  65.4  0.0 |
| **Sturdy plunger during use (%)**  Better with the Somatuline Autogel syringe  Same rating for both syringes  Better with the Lanreotide Pharmathen syringe | 92.6  5.9  1.5 | 84.6  15.4  0.0 |
| **Confidence that there is a low risk of needle-stick injuries (%)**  Better with the Somatuline Autogel syringe  Same rating for both syringes  Better with the Lanreotide Pharmathen syringe | 39.7  35.3  25.0 | 34.6  42.3  23.1 |
| **Confidence that there is a low risk of contamination of the syringe from preparation to injection (%)**  Better with the Somatuline Autogel syringe  Same rating for both syringes  Better with the Lanreotide Pharmathen syringe | 58.8  27.9  13.2 | 76.9  23.1  0.0 |
| **Confidence that the syringe needle appears patient friendly (%)**  Better with the Somatuline Autogel syringe  Same rating for both syringes  Better with the Lanreotide Pharmathen syringe | 32.4  23.5  44.1 | 42.3  23.1  34.6 |
| **Easy to teach others how to use (%)**  Better with the Somatuline Autogel syringe  Same rating for both syringes  Better with the Lanreotide Pharmathen syringe | 73.5  20.6  5.9 | 69.2  30.8  0.0 |
| **Number of attributes per nurse rated better with the Somatuline Autogel syringe**  Mean  Standard deviation  95% Confidence interval | 7.6  2.81  6.6, 8.0 | 7.3  2.47  6.3, 8.3 |
| **Number of attributes per nurse rated better with the** Lanreotide **Pharmathen syringe**  Mean  Standard deviation  95% confidence interval | 1.3  1.71  0.8, 1.7 | 0.7  0.83  0.4, 1.1 |

^a^The difference in ratings of the Somatuline Autogel syringe and Lanreotide Pharmathen syringe was calculated as the rating score for each attribute for Somatuline Autogel syringe minus the rating score for each attribute for Lanreotide Pharmathen syringe. The class described as better rating with Somatuline corresponds to a difference in ratings of Somatuline Autogel syringe and Lanreotide Pharmathen syringe > 0. The class described as same rating for both syringes corresponds to a difference in ratings of Somatuline Autogel syringe and Lanreotide Pharmathen syringe = 0. The class described as better with the Lanreotide Pharmathen syringe corresponds to a difference in ratings of Somatuline Autogel syringe and Lanreotide Pharmathen syringe < 0.

**Supplementary Fig. 1a** Ranking of attributes by importance in Europe


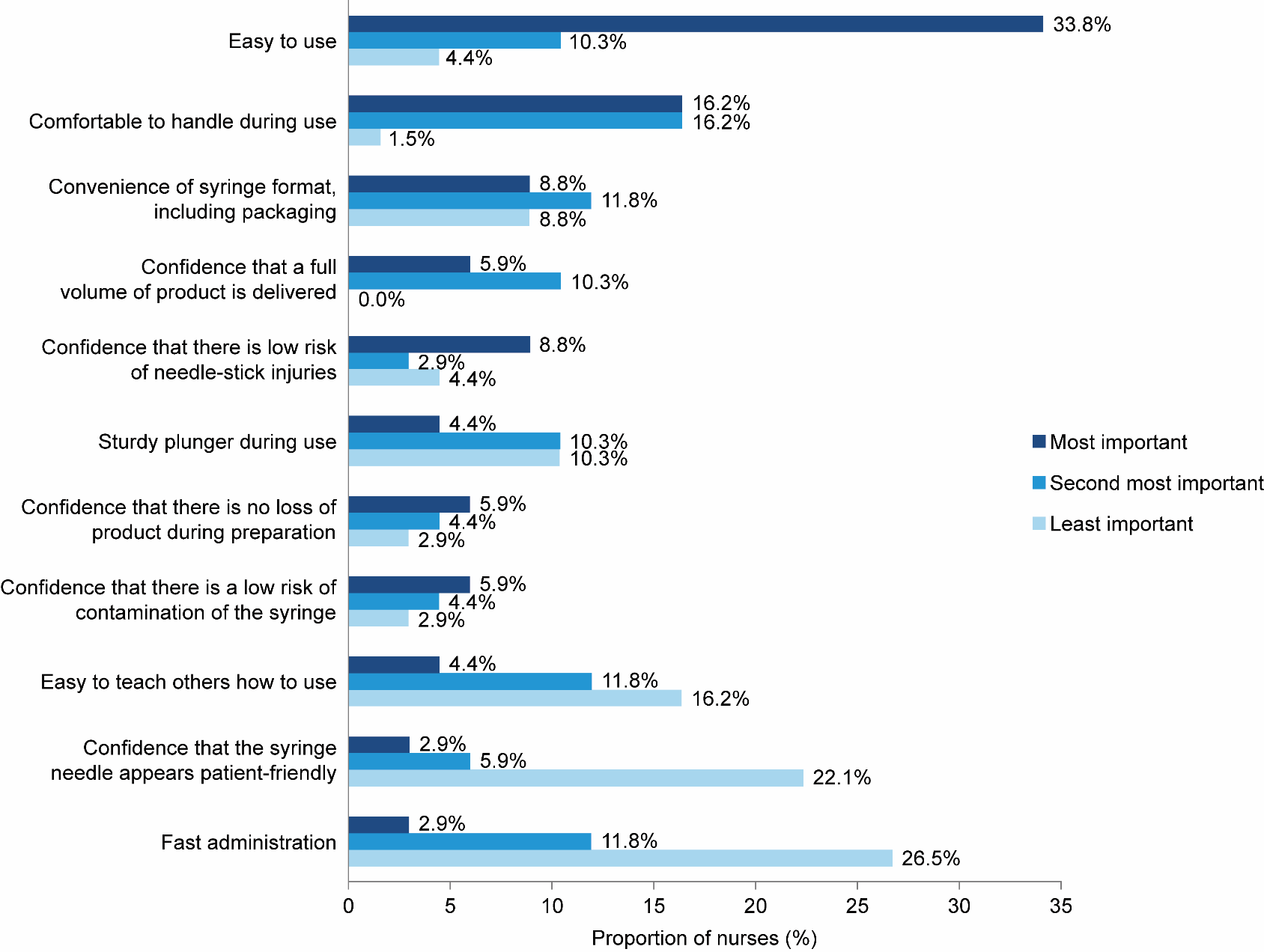


**Supplementary Fig. 1b** Ranking of attributes by importance in US

**
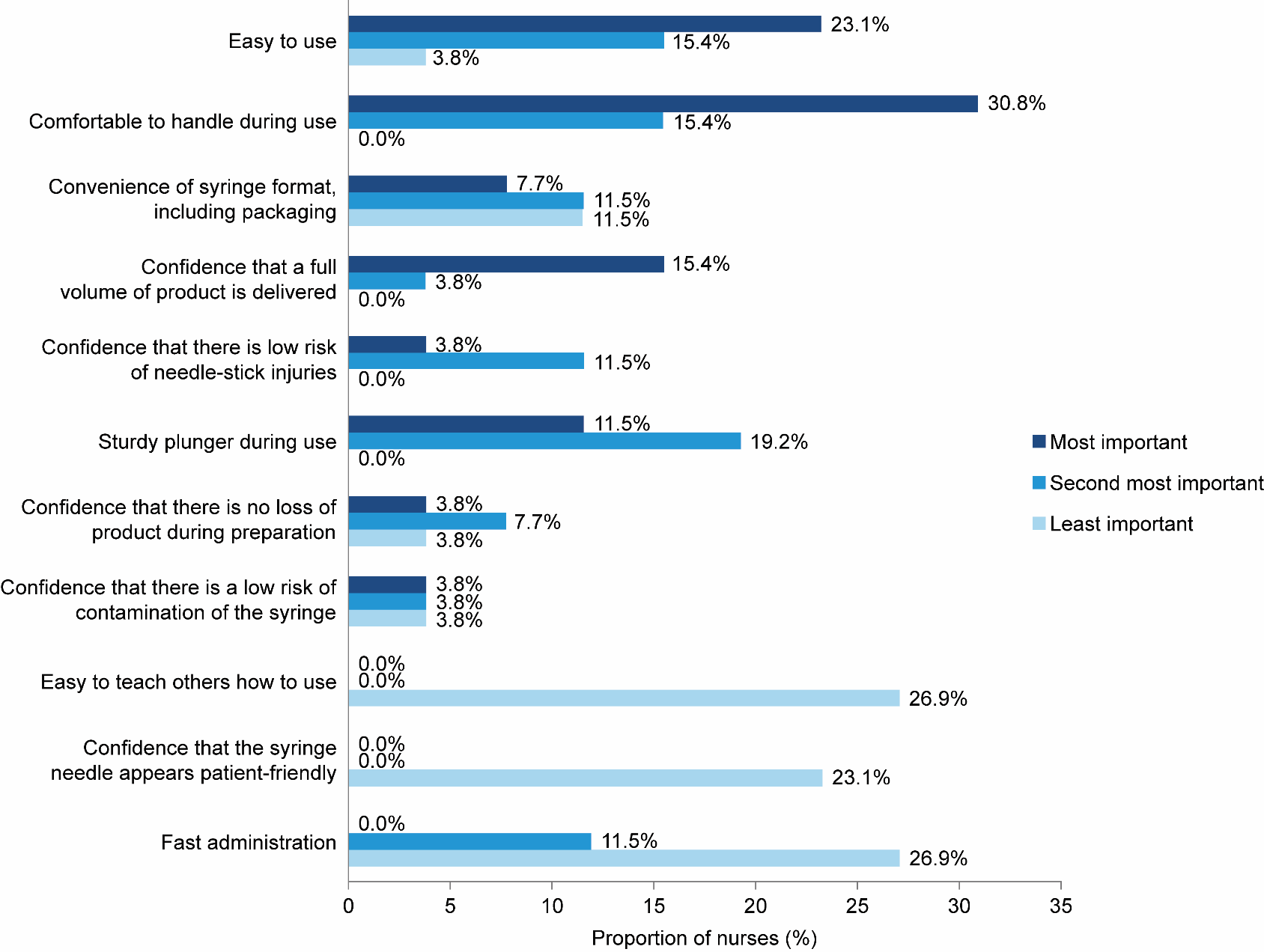
**
